# How School Nurses Experience Their Work with Schoolchildren Who Have Mental Illness – A Qualitative Study in a Swedish Context

**DOI:** 10.5539/gjhs.v6n4p1

**Published:** 2014-03-17

**Authors:** Fikrije Dina, Zada Pajalic

**Affiliations:** 1 Kristianstad Municipality, Kristianstad, Sweden; 2 Med Sci Kristianstad University, Kristianstad, Sweden

**Keywords:** experiences, schoolchildren, school nurse, mental illness, qualitative research

## Abstract

**Background:**

Reports from research have shown that mental illness has increased dramatically in recent years and is currently one of the biggest problems among Swedish children and adolescents.

**Aim:**

The aim of this study was to describe how Swedish school nurses experience their work with schoolchildren who have mental illness

**Method:**

Data were gained by individual interviews with school nurses (n = 10) and were analyzed by using manifest qualitative content analysis.

**Results:**

The results of the study showed that school nurses used various tools when working with schoolchildren who have mental illness. The working tools were regular health talks, motivational interviewing, individual counseling, family counseling, creating trust, and affirming the child’s confidence.

**Conclusion:**

Results of the study demonstrate the need for further research on schoolchildren’s experience of getting help and support from the school nurse.

## 1. Background

Mental illness currently is one of the biggest problems among Swedish children and adolescents (Johansson & BRIS, 2012). Mental illness is a subjective experience of not feeling well. You may feel anxious, depressed, or stressed (Clausson, Kohler, & Berg, 2008b; Johansson & BRIS, 2012). Mental illness is defined as two main categories subdivided into internalized (inward) and externalized (aggressive) problems (Petersen et al., 2010). Internalizing problems include symptoms such as anxiety, worry, depression, and physical problems, while externalizing problem behaviors include poor self-control, defiance, impulsivity, and aggression (Honeyman, 2007; Thompson, 1977).

The studies from 2012 show that the mental illness has increased dramatically among children and adolescents in recent years (Johansson & BRIS, 2012). Further it was shown that schoolchildren who are unable to stop their momentum to attack their classmates, teenagers without zest for life, and young people seeking emergency help due to palpitations and panic disorder (National Board of Health and Welfare & Development Center for Children’s Mental Health, 2009). Swedish school nurses reported in a study that psychosomatic health problems such as stomach pain, headaches, and anxiety were the most common reasons for schoolchildren’s spontaneous consultation with a school nurse, events especially common in girls (Clausson & Berg, 2008; Clausson, Eva, Petersson, Karin, & Berg, Agneta, 2003; Clausson et al., 2003; Clausson et al., 2008a; Clausson et al., 2008b).

Mental illness can prevent a child from assimilating teaching, engaging in leisure pursuits, and enjoying socializing with peers (National Board of Health and Welfare, 2004b). Mental health problems in childhood and adolescence are often precursors of continued problems in adulthood (National Board of Health and Welfare, 2004a; National Board of Health and Welfare & Development Center for Children’s Mental Health, 2009, 2010a, 2010b). The report *View All of Me* (Johansson & BRIS, 2012) showed that mental health problems in children often lead to consequences like deteriorating school performance, difficulty sleeping, feelings of loneliness, anxiety, self-hatred, shame, fear, guilt, self-destructive behavior, eating disorders, suicidal thoughts, difficulty controlling anger, and shying away from interacting with others (Alfvén & Alfvén, 2006; Cullberg, 2006).

Student health has the mission to organize and provide health care for children and adolescents in school health services. All students in Sweden are entitled to student health, whether they attend a public or private school (National Board of Health and Welfare & Development Center for Children’s Mental Health, 2010b); student health is offered and implied that they take up the offer. There is thus a risk that parents will reject this offer (National Board of Health and Welfare, 2004b). The new Education Act (Ministry of Education, 2010a), which came into force on 1 July 2011, replaces the concept of school health with student health and states in § 25 that there should be student health services for students in preschool classes, elementary school, compulsory school, Sami school, special school, secondary school, and upper secondary school. Student health includes medical, psychological, psychosocial, and socio-educational services. Student Health will be concerned primarily with prevention and health promotion. Pupils’ progress toward the goals of education should be supported. For medical, psychological, and psychosocial interventions, students must have access to a school physician, school nurse, psychologist, and social worker (Ministry of Education, 2010a, 2010b).

The mental health among schoolchildren thus has declined dramatically in recent years, as discussed above, and is currently one of the biggest problems among Swedish children (Johansson & BRIS, 2012). Because only a few empirical studies based on Swedish school nurses’ scientific perspective have touched on the subject, and because we need to gain a deeper understanding and greater knowledge of this problem, we considered it valuable to study the school nurse’s experience of working with schoolchildren’s mental health issues. The aim of this study was to describe how Swedish school nurses experience their work with schoolchildren who have mental illness.

## 2. Method

### 2.1 Design

The study has used descriptive design with a qualitative approach (Polit & Beck, 2013). The first characteristic of qualitative researchers is that they often put together a complex collection of data obtained from a variety of sources and use different methods. This semi structured in depth research interview seeks to obtain uninterpreted descriptions (Kvale, 2007). The interviewed informant describes as accurately as possible what she is experiencing and feeling and how she acts. The key is to obtain comprehensive descriptions that reproduce the qualitative diversity and all the differences, variations, and complexities of an event (Kvale, 2007; Kvale & Torhell, 1997). For the present study are semi structured in depth interviews used.

### 2.2 Context and Participants

The survey was conducted in two medium-sized municipalities, located in southern Sweden, with a total of 62,413 inhabitants. The study group was nurses working in primary schools within the school health systems and includes students from preschool to grade six. The characteristics of these schools are that they are divided into primary and middle schools and are located in elementary schools. Another important feature is that all the schools are owned by municipalities. Although the distances were far between the various schools, in some schools schoolchildren of Swedish descent dominated, while in other schools children of foreign origin dominated. Inclusion criteria for participation in the study specified nurses who have specialist training as district nurses or in health care for children and young people and have worked at least one year as a school nurse. Both female and men who work as school nurses were included in the study. The author interviewed ten nurses. Of these, all were female. Their professional experience as nurses ranged from 1.5 years to 23 years, with an average time of 10 years spent as a school nurse. The school nurses were between 38 and 61 years old; the average age was 46 years. Eight nurses had specialized training in health care for children and adolescents and two nurses had specialized training as district nurses. The number of schools and students that school nurses were responsible for ranged from two to seven schools and to between 300 to 725 students.

The purposive selection of informants in this study is appropriate (Polit & Beck, 2013). We consciously chose purposive sampling technique of people who would be most useful in the study. The selection was designed: (1) to find people who are appropriate to find some degree of interest in their work (2). An information letter explaining the purpose of the study was sent to two business managers for school nurses in two different municipalities in the southern part of Sweden to get written permission to conduct the study and to gain access to potential informants (nurses). One of two operations managers gave the author permission to conduct the study. The operations manager who did not give the author permission to conduct the study gave as a reason that school nurses’ lack of time to participate in it. This led the author to broaden the application. Another operations manager in another municipality was asked for written authorization to conduct the study, and he gave the author permission. After the author received a written authorization from business managers to conduct the study, school nurses were contacted by telephone with and were informed about the purpose of the study and asked if they were interested in participating. In one municipality contacted by the telephone, seven of thirteen nurses met the inclusion criteria and agreed to participate. The loss was six nurses; two of them met the inclusion criteria but were unable to participate due to time constraints, one of them did not meet the inclusion criteria, and three of them did not respond at all, despite repeated phone calls and voice messages by phone. In the second municipality we contacted two nurses over the phone; both met the inclusion criteria and both agreed to participate, so the loss was zero. All nurses who agreed to participate were sent an information letter with written information and an invitation to participate in the study. The author booked the time and place of the interview in consultation with the school nurses who chose to participate in the study.

### 2.3 Data Collection Method

Data collection took place from October 2012 to January 2013. Data was collected through individual interviews based on an interview guide constructed with semi-structured questions and supplementary questions based on the study’s purpose and questions (Polit & Beck, 2013). The items under investigation and the order in which they were addressed during the interview contained background questions about each school nurse’s gender, age, education, work experience, and responsibility for the number of pupils/schools, among other questions. Specific questions asked were *(1). Describe in as much detail and as much as possible how to work with schoolchildren who have mental illness. Supplementary questions included: What do you mean? Can you elaborate on that? Do you have anything more to add?* A pilot interview was conducted with the result that the questions were tested on a school nurse so the author could ensure that the proper issues were addressed and that the answers responded to the purpose of the study. The pilot interview showed that no changes in the interview guide were needed. All interviews were conducted in the school nurse’s office. The school nurses gave their approval by signing an informed consent form. The interviews were recorded on tape. The interviews were transcribed verbatim immediately after the interview so that nothing important was missed, which means that even pauses, laughs, or coughs were recorded. The length of the interviews varied between 15 and 25 minutes.

### 2.4 Data Analysis

Qualitative content analysis according Graneheim and Lundman (2004) was used to analyze the transcribed interviews. The focus in content analysis is to describe variations by identifying similarities and differences in text content. Differences and similarities are expressed in categories and themes at different levels of interpretation. In every text there is a manifest content and a latent message. The manifest content of the text closely reveals the content and is expressed on a descriptive level in terms of categories. Manifest analysis was chosen by the author to analyze the text of the transcribed interview area where the aim was to describe the school nurse’s experience. The transcribed interviews were included in the analysis as an analysis unit (unit of analysis). The transcripts were read through several times by the first author (FD). First, a general, transparent reading was performed to get a sense of the whole; the next step was a reading to aim for a more thorough and in-depth understanding with the intent of getting ideas for further analysis with the purpose of the study as the basis. The text was then split into domains, namely, calls to identify and cooperate. A domain is part of the text that relates to a specific area and serves as a rough structure that is possible to identify with a low degree of interpretation. Meaningful units (meaning units) were picked out by the study’s end. A meaningful unit may consist of words, sentences, and paragraphs of text that are related in content and context. During the analysis, the process was condensed and abstracted. Abstraction is a process that makes the text shorter and therefore more manageable, while the core content is preserved and nothing of significance disappears. Then the condensed text was abstracted, provided with codes, and subsequently merged into subcategories and categories.

### 2.5 Ethical Considerations

The study was performed in accordance with the Helsinki Declaration (World Medical Association, 2001) (Saif, 2000). All participants gave their informed consent to participate in the study after having been presented with detailed information about the study and their own participation. They were also informed that they had the right to terminate their participation at any time without consequences.

## 3. Results

The result of ten interviews with school nurses reported on the school nurse’s work tools; the school nurse’s experience identifying mental illness; and the school nurse’s experience of cooperation with other professionals, agencies, and parents.

### 3.1 The School Nurse’s Working Tools

The results of the study showed that the school managers used various work tools when dealing with schoolchildren’s mental health. The school nurse used regular health talks, motivational interviewing, individual counseling, and family counseling. An important part of the school nurse’s job was to create a trust and affirm the child’s confidence.

### 3.2 Health Talk

The results of the study showed that health talks were an important tool for the school nurse. During the health talks school nurses use of “bear cards” that help the child to express different emotions.

“*I have children who come to call, and then it can be, we use something called “bear short”; it’s nice pictures of koala bears expressing different emotions and there is no text, and the children can then speak from those cards: How do you do? How do you think your family feels or how is it with your classmates? … And it can be a good way to start*.” It also emerged that in a healthy conversation, the school nurse could use the power card describing the various characteristics of the child and use a self-assessment questionnaire to capture the child’s emotional state. “*… There are also cards … A strength card has various properties the child can pick out for himself, describing himself from these cards …”*

### 3.3 Motivational and Individual Interviews

Motivational and individual interviews were relevant in the school nurse’s work. Motivational interviewing was dedicated to promoting a positive lifestyle and a healthy lifestyle. “*Motivational interviewing’s like this … technique that is very effective … It could … be about if you are depressed and dejected that you actually can find activities that are good … to find, start to go and swim or go for a walk, doing physical things can make you feel physically better…”* In individual interviews the child could go to the school nurse and tell her how he or she feels.

### 3.4 Family Counseling

The study’s results showed that working with the whole family and involving parents was usually important in dealing with mental illness in schoolchildren. The school nurses stressed that when it came to children from grade one to grade six, it was especially important to talk to the parents and to involve them when it was appropriate. “*And then it’s the parents--so it is about the smaller children now … we cannot do anything without the parents, without parental consent … you need them on the train the whole the time…”*

### 3.5 Creating Trust

Many nurses felt that a quiet environment, security, and taking the time to listen led the children to find it easier to talk about their concerns and thus created trust in the school nurse. “*… For my part, I think it feels like I need to give them peace of mind so that they might dare to open up a bit more about what it is that is their concern.”*. “*…Be responsive if the child starts to talk, that you then take the time to listen… Most often it’s the person who knows the child the best so they can open themselves…”*

### 3.6 To Affirm the Child’s Self-Confidence

Attempts to strengthen the child’s self-confidence were demonstrated in the study. The school nurses could feel powerless at times because it was hard for them to make big changes. Then it was important to support and motivate the children to find their value in life and to equip them for the future. “*These gray area kids … You may try to run and motivate them to find their own value in life, and are they prepared for the future, that they should make their own wise choices.”* The school nurses found working in groups valuable for strengthening the child’s self-confidence through values clarification. “*Something that I think is good is working in groups in class, sometimes having different valuation exercises where you stand up for your opinions in front of others and … We take up sensitive issues in class and in groups and how to do … and I think they may be strengthened by such exercises*.”

### 3.7 The School Nurse’s Experience in Identifying Mental Health Issues

Results of the study showed that all nurses had identified mental illnesses among schoolchildren such as agitation, anxiety, depression, and other neuropsychiatric disorders. Their experience in the profession and their feelings when a child is hurting them self physically helped them to be able to identify mental illness quickly in a child. The identification could be made in conjunction with a healthy conversation. Even when the child visited the school nurse several times and complained of little concerns such as sore feet, sore hands, and a headache or the child told me as well that for example he heard voices or made an explicit suicide threat. “*… Many will hit themselves and tell. then when I make the right health calls, that’s where I catch problems more efficiently, so to speak…”* The parents could call the school nurse and tell the nurse they are worried about their children. Classroom teachers turned to the school nurse if they received signals that a child was not feeling well. Classmates, especially those in grades five and grade six, turned to the school nurse and told her they were worried about a friend they had noticed was not himself, was feeling sad, and more. Identification of mental health problems among schoolchildren led the school nurses to refer the child to the emergency room, the children’s hospital, or to child and youth psychiatry for further investigation. *“Anorexia … where I know that the parents come to me and said, it does not look good; then I went and picked up the girl--no, it was not good. She had a weak pulse, her hands were cold, and I could not get any blood pressure; she was pale, tired, very rundown. Then I sent her to the children’s emergency room, where they made a medical examination, and then she came to child and youth psychology.”*

### 3.8 The School Nurse’s most Difficult Experiences in the Identification of Mental Illness

The results of the study showed that some nurses felt it was difficult when they were informed by the teachers that a child had a mental illness. *“…* *Then it is most difficult when I get information … that’s if I get it … if the teacher comes to me and tells me that I’m so worried about this child, can you do something? For a while, then you have to think about that it is I who will be taking care of this.”* The school nurses found it difficult when the parents did not show understanding. Other nurses found it difficult to call home to the parents and say that their child has expressed that he or she does not feel well. *“It can be difficult to call home to the parents and say that their children have expressed to me that they do not feel well … it’s probably one of the hardest…”*. Some nurses expressed that they felt frustrated not being able to make big changes and that there was not enough time. “*The hardest thing… the most difficult thing… it’s that I’m not enough, it’s that I do not have time, it’s the very worst thing that I think of myself…”*

### 3.9 The School Nurse’s Experience of Cooperation with Other Professionals, Agencies, and Parents

One of the study’s results showed that one task with mental health problems among schoolchildren was the work with many different professions and agencies, such as school counselors, teachers and principals. Cooperation with child and youth psychiatry came when the school nurses sent a referral for a child who had a mental illness. Social Services came into play in connection with the notification that a child had been badly treated. Cooperation with Child House came when the nurse suspected that a child had been subjected to a violation by any adult or parent; the child was sent there and all the staff got there so that the police and the pediatrician could examine the child and an x-ray was done at the hospital. The collaboration worked fine; sometimes secrecy can cause problems, but the parents were with them so it worked mostly well. Cooperation with the parents was an important part of the work.

### 3.10 Cooperation with School Counselor

The school nurses work closest with the school counselor. The contact between the school nurse and school counselor was very close, despite different vocational skills, but they shared a certain sphere, they juggled their duties and skills with each other and greatly benefited from each other’s expertise. Some schools had school counselors in place, at other schools counselors were available a few days a week, and at other schools there were no school counselors at all. In schools where counselors were not available, school nurses felt lonely and overworked; in situations where there was a counselor available, they did not have to juggle when a problem with a child arose. School nurses had more children visit with mental illness. *“… The cooperation that I have such has been with the school counselor it is the most often between us, it’s nothing that we’re talking about with the teams … but I almost think that the best collaboration is that it works directly between the school nurse and the school counselor, who do not go through the team “*.

### 3.11 Cooperation with Student Health Team

All nurses experienced working with the student health team. The student health team included the school nurse, school counselor, principal, the special education team, social educator, psychologist, and sometimes even teachers and speech therapists. The student health team met and discussed children who feel poor. The discussion about a child who was feeling poorly concerned how to proceed, how to help the child, how to help the family, whether help from child psychiatry and social services was needed. It was helpful when the student health team met frequently, at least a couple times a month. *“… It is interesting sometimes when you are sitting in the student health team with the principal, counselor, special education teacher, psychologist and speech therapist at times and that they have different skills that they can use to look at the case from different angles… it’s rewarding that it goes beyond school nurses and pediatric nurses and offers a multifaceted view of the students…”*

### 3.12 Cooperation with Parents and Other Parties through Meetings

Among the study’s results, it appears that school nurses had experience working with parents and other agencies also through meetings. People from school, child and youth psychiatry, social services, children’s rehabilitation, and parents were invited to a meeting. The school nurses experienced such meetings as very important in part because you could break confidentiality and in part because the different parties and their parents were working toward the same goal, namely, to help the child. “*We can have meetings … it’s then when the parents want you to call a meeting, everyone involved … different authorities and different health care interventions and then writes the parents in a paper that secrecy may be lifted by then I can speak freely … and it may be that say Child and youth psychiatry and it may be Social Services are there and … everyone attending as well as schoolteachers and stuff … and that is to work forward … so it’s a really great way to have a meeting if there are many people involved with a student … “*.

## 4. Discussion

### 4.1 Discussion of Results

Results of the study showed that school nurses used various tools when working with schoolchildren who have mental illness. It was revealed that all nurses had been through and identified the mental health of schoolchildren. It also emerged that in the work of mental health problems among schoolchildren it is important to work with many different professionals, agencies, and parents (Barnard & Neal, 1977; Browne, Cashin, & Graham, 2012) (Kim, 2010). According to the National Board (2004b), health talks by school nurses developed a special form of support and a health-promotion approach, based on individual strengths and weaknesses, which is in line with the results of the study. The results of the study showed that the school used various tools when working with schoolchildren’s mental health in which health talks were an important tool (Browne et al., 2012). During the health talks, school nurse used bear cards, for example, that helped the child express various emotions. Another study (Golsäter, Sidenvall, Lingfors, & Enskär, 2011) described the schoolchildren’s health as an opportunity to get the support of the school nurse. Healthy speech was perceived as an opportunity to establish contact with the school nurse and try to find out if she was someone to be trusted or not (Lundquist, 2008).

Working with the whole family and involving the parents was often important in mental illness in schoolchildren, the study’s results showed. This is supported by several studies (Clausson & Berg, 2008; E. Clausson et al., 2003; E. K. Clausson et al., 2008a, 2008b). The school nurse listened to the child and then contacted the parents and told them about the situation and asked them to come to her office to discuss the situation (Pidgeon, 1985; Pryjmachuk, Graham, Haddad, & Tylee, 2012). By meeting together with the child, the parents, and the school nurse, the school nurse could help the child to express the problem in words (Morberg, Lagerstrom, & Dellve, 2012). Another study highlighted the importance of therapeutic conversations with families who had children who suffered from mental illness (Thompson, 1977). Therapeutic conversations seemed to trigger a healing process, including affective, cognitive, and behavioral changes in family function where the families became more and more aware of their own resources and capabilities to manage their health problem (Maenpaa & Astedt-Kurki, 2008). The meetings led to feelings of relief; families felt affirmed and created their own solutions (Clausson et al., 2008).

In was shown that the most common way for the nurse to pay attention to mental health problems among the schoolchildren was through health talks, which is in line with the results of the study that stated that the identification of mental health problems among schoolchildren could be accompanied with a healthy conversation (National Board of Health and Welfare & Development Center for Children’s Mental Health, 2009). A study by Clausson, Kohler, and Berg (2008) showed, however, that the health checks and health talks were assessed and declared a common basis for assessing the physical health of the schoolchildren and less commonly to assess mental health. Among the study’s results was the revelation that school nurses could identify mental illness in schoolchildren even when the child visited the school nurse several times and complained of little concerns like sore feet, sore hands, and a headache. Schoolchildren’s spontaneous visits to the school nurse seemed to be more common and were warranted to assess schoolchildren’s mental health. Study results also showed that the identification of mental health problems among schoolchildren led to school nurses referring the child to the children’s emergency room, a children’s clinic, or child psychiatry for further investigation. This is supported by a study by Moberg and colleagues (2012), which identified school nurses as “gatekeepers,” identifying first-line problems and contacting parents and other professionals if necessary.

Among the study’s results was the demonstration the work with mental health problems among schoolchildren was part of school nurse’s duties, including collaboration with many different professionals, agencies, and parents. It was revealed that school nurses had experience in working with parents and other parties through meetings that were very important because they all were working toward the same goal, namely, to help the child. Moberg and colleagues (2012) described in their study of school nurses’ consideration of themselves as advocates for the schoolchildren; when a child appeared to be suffering from mental illness, she could assess the child’s school situation and the school environment, talk to counselors, psychologists, teachers, and most importantly, involve the child’s parents. In a study by Pryjmachuk and colleagues (2012), parents were seen as important and needy when a schoolchild showed mental illness, and often they wanted immediate change in their child’s behavior. Another study showed that parents were willing to participate in their children’s health examinations and they wanted nurses to invite them often. When the parents were invited, it offered them an opportunity to express their own views about the child’s health. Parents experienced such meetings as relaxed and friendly (Maenpaa & Astedt-Kurki, 2008).

## 5. Conclusion

In summary, the school nurse’s experiences of identifying mental health problems among schoolchildren as well as their experiences working with other professionals, agencies, and parents refers to the nurse’s cognitive, behavioral, and social aspects of professional action. The results of the present study show that the school nurse have an important role in reasoning, making decisions, transferring knowledge into action, putting available knowledge into practice and taking certain actions. In conclusion, this study shows that the school nurse has a central role in the work of schoolchildren’s mental health. Results of the study raise concerns about further research on schoolchildren’s experiences getting help and support from the school nurse.
